# Whole genome sequencing and analysis of the weed pathogen *Trichoderma polysporum* HZ-31

**DOI:** 10.1038/s41598-024-66041-w

**Published:** 2024-07-02

**Authors:** Yushan He, Haixia Zhu

**Affiliations:** 1 Academy of Agriculture and Forestry Sciences, Qinghai University, Xining, 810016 China; 2 State Key Laboratory of Plateau Ecology and Agriculture, Qinghai University, Xining, China; 3 Key Laboratory of Agricultural Integrated Pest Management of Qinghai Province, Xining, China

**Keywords:** Whole genome, *T. polysporum* HZ-31, Weeds, Key genes, Microbiology, Molecular biology

## Abstract

In order to resolve the key genes for weed control by *Trichoderma polysporum* at the genomic level, we extracted the genomic DNA and sequenced the whole genome of *T. polysporum* strain HZ-31 on the Illumina Hiseq platform. The raw data was cleaned up using Trimmomatic and checked for quality using FastQC. The sequencing data was assembled using SPAdes, and GeneMark was used to perform gene prediction on the assembly results. The results showed that the genome size of *T. polysporum* HZ-31 was 39,325,746 bp, with 48% GC content, and the number of genes encoded was 11,998. A total of 148 tRNAs and 45 rRNAs were predicted. A total of 782 genes were annotated in the Carbohydrase Database, 757 genes were annotated to the Pathogen-Host Interaction Database, and 67 gene clusters were identified. In addition, 1023 genes were predicted to be signal peptide proteins. The annotation and functional analysis of the whole genome sequence of *T. polymorpha* HZ-31 provide a basis for the in-depth study of the molecular mechanism of its herbicidal action and more effective utilization for weed control.

## Introduction

Weeds are one of the major constraints on crop production, and the weed flora is diverse. It consists of a variety of perennial and annual grasses, broadleaf weeds, and sedges, which includes both parasitic and invasive weed species. Weed control is usually carried out using chemical herbicides and tillage. However, the overuse of these two control strategies poses significant challenges to agricultural production and ecology^1^. As the source of the Three Rivers, protecting the ecological environment in the Tibetan Plateau region is a top priority. This region also has special ecological characteristics, such as high altitudes, severe cold, strong radiation, and drought conditions^2^, resulting in a very low environmental carrying capacity^3^, and making the ecological environment is extremely fragile and vulnerable to damage. The extensive use of chemical herbicides has made the Tibetan Plateau region prone to ecological pollution, weed resistance and other problems. Reducing the amount of chemical herbicides used in response to the special ecological conditions of the region can effectively reduce the pollution of the ecological environment and increase crop yields. Many microorganisms and their metabolites have herbicidal activity, and they have received more attention in the field of biocontrol research in recent years^4^. Microbial herbicides developed using microbial metabolites, especially phytopathogenic toxins, are usually safe, environmentally friendly, highly effective and have multiple target sites of action, so they can achieve green and efficient control and disrupt the development of weed resistance^5^.

Xylomycetes are soil-borne filamentous fungi that are widely used as a source of biocontrol agents in agriculture^6^. They various have effective antagonistic mechanisms^7^, such as fungal parasitism^8^, antibiotics^9^ or competition against plant pathogens^10^ and nematodes^11^. Some of the active products of *Trichoderma* have also been shown to exhibit herbicidal activity in recent years. Javaid et al.^12^ demonstrated that fermentation filtrates of *Trichoderma harzianum* Rifai, *Trichoderma pseudokoningii* Rifai, *Trichoderma reesei* Simmons and *Trichoderma viride* Pers. have herbicidal activity against the wheat field weeds *Phalaris minor* L. and *Rumex dentatus* L. Yin et al.^13^ demonstrated that harzianum A and B from *Trichoderma breviccompacactum* showed efficient weed control potential at low concentrations against *Brassica chinensis*, an herbaceous plant of the Cruciferae family. Moura et al.^14^ demonstrated that methanolic extracts from *Trichoderma spirale* affected the photosynthesis of *Senna occidentalis* and *Ipomoea grandifolia*, an herbaceous plant of the genus *Ipomoea*, thereby exerting a toxic effect on these weeds. While searching for potential herbicidal compounds, our laboratory found a strain of the weed causal agent *T. arvense* var. setosum at the stem base of *Cirsium arvense* var. setosum that showed highly efficient inhibition of weeds such as *Avena fatua* L., *Chenopodium album* L., *Polygonum lapathifolium*, and others. *Trichoderma polysporum* HZ-31^15^, which can produce a variety of active substances such as 1,8-propanediol-o-xylene, 2,3-dihydroxypropyl propionate, and others^16^, is a kind of mycobacterial resource with good potential for utilization in weed biological control. At present, the whole genome sequence of *T. polysporum* has not been reported in the literature, and the use of the whole genome to mine the genes related to the synthesis of pathogenic and bioactive substances of *T. polysporum* has not been reported.

Sequencing the genomes of weed-producing defensive fungi and using bioinformatics methods to search for the disease-causing genes and genes related to synthesis of bioactive substances or signaling pathway components of weed pathogens is an effective method for studying the pathogenesis of weed pathogens. In this study, we sequenced the genome of *T. polysporum*, performed genome sequence analysis and gene function annotation to identify pathogenicity-related genes at the genome-wide level, searched for genes related to secondary metabolite synthesis in the standard databases and performed a systematic analysis. The results of these analyses provide a scientific basis for the in-depth study of the pathogenesis of *T. polysporum* in weeds.

## Material for testing

### Test strains

*Trichoderma polysporum* HZ-31 was provided by the Key Laboratory of Integrated Management of Agricultural Pests of Qinghai Province and was stored in the General Microbiology Center of China Microbial Culture Preservation Management Committee (CGNCC No.12867).

## Methods

### Strain culture

The laboratory strain preserved in the slant medium of *T. polysporum* HZ-31 was transferred into PDA medium, using a sterile perforator with a diameter of 8 mm at the edge of the colony to hit the mycelium blocks, and was then inoculated on an aseptic operating table in 250 mL of sterile PDB liquid medium. Each bottle of inoculation included 5–8 mycelium blocks, and was cultured at 25 ℃ on a shaking bed at 180 rpm for 5–7 days. After filtering with three layers of sterile gauze, the mycelium and spores were collected, washed three times with sterile water, frozen in liquid nitrogen and prepared for use.

### Extraction of genomic DNA (gDNA)

Genomic DNA was extracted using the Fungal Genomic DNA Extraction Kit (purchased from Beijing Solaibao Technology Co., LTD.). The integrity of the DNA was determined using 1% agarose gel electrophoresis, and the concentration and purity of the DNA were determined using Thermo Qubit 4.0 (purchased from Life Technologies).

### Library construction, sequencing, data quality control and assembly

The whole genome DNA of *T. polysporum* HZ-31 was constructed and sequenced by Sangong Bioengineering (Shanghai) Co. The genomic DNA was sequenced using the Illumina II sequencing platform. After library construction, the library size was determined by 2% agarose gel electrophoresis, and the library concentration was measured by a Thermo Qubit 4.0 fluorescence quantification instrument.

The raw image data files obtained by Illumina Hiseq were converted into raw sequenced reads by CASAVA Base Calling analysis. The raw data quality values and other information were determined, and the quality of the sequencing data of the samples was evaluated visually using FastQC. The raw data wasfiltered using Trimmomatic 0.36, which included removing the following: sequences with N bases; splice sequences in reads; low-quality bases (Q-value < 20) starting from the 3′ to 5′ direction of reads; low-quality bases (Q-value < 20) starting from the 5′ to 3′ direction of reads; bases with quality values below 20 in the tails of the reads using the sliding window method (window size of 5 bp); andcases where the read itself along with its paired read had a length of less than 35 nt.

The second-generation sequencing data wasspliced using SPAdes 3.5.0, which first corrects the sequence errors of the original sequence, then assembles the sequences by multiple Kmer values, and finally synthesizes the assembly results of each Kmer value to obtain the best results. Then GapFiller 1.11 was used to complement GAP on the contig obtained from splicing, and finally PrInSeS-G 1.0.0 was used for sequence correction to correct the editing errors and the insertion-deletions of small fragments during splicing.

### Gene element prediction

GeneMark 1.10 was used for gene prediction of the assembly results, with tRNAscan-SE for tRNA, RNAmmer for rRNA, and Rfam for snRNA, while RepeatModeler was used for the Denovo prediction of repetitive sequences of the assembly results. RepeatMasker was then used to find the position and frequency of occurrence of each type of repetitive sequences on the genomic segments.

### Gene function annotation

The protein sequences of the predicted genes were aligned with the NR, SwissProt, TrEMBL, COG, PFAM (http://pfam.xfam.org/), and CDD (https://www.ncbi.nlm.nih.gov/cdd/) databases to obtain protein functional annotation using NCBI BLAST+ 2.2.28 software. GO (Gene Ontology) functional annotations were obtained using the SwissProt and TrEMBL databases, and KEGG (Kyoto Encyclopedia of Genes and Genomes) annotations were obtained using KAAS 2.21 software.

### Analysis of disease-causing and secondary metabolite-related genes

The gene set protein sequences were aligned to the CAZy database using HMMER3 3.1b1 to obtain their corresponding carbohydrate-active enzyme annotation information. The screening condition was E-value < 1e−5. The gene protein sequences were aligned with the PHI-base database using BLAST to combine the annotation information of the genes and their corresponding pathogenic host interactions and obtain the final annotation results. Secondary metabolite synthesis gene clusters in strain PA-2 were predicted using the antiSMASH 3.0 online tool (https://fungismash.secondarymetabolites.org). SignalP 4.1 software was used to predict the possible signal peptides of *T. polysporum* HZ-31.

## Results and analysis

### Genome assembly

The total base length of all contigs of *T. polysporum* HZ-31 was 39,325,746 bp, with an average GC content of 48%, including 891 contigs with a total average length of 44,136.64 bp, and an N50 value of 155,640 (Table 1). The results of the genome sequencing of *T. polysporum* HZ-31 were uploaded to NCBI, with accession number PRJNA941260.

**Table 1:** Assembly results of *Trichoderma polysporum* HZ-31.

Essential feature	HZ-31
Sum of base lengths of all contigs	39,325,746
Sum of N-containing bases and fuzzy bases	868
Maximum contig length	750,229
Average contig length	44,136.64
Number of contigs	891
N50	155,640
GC ratio	0.48

### Gene element predictions

#### Coding gene predictions

**Figure 1:**
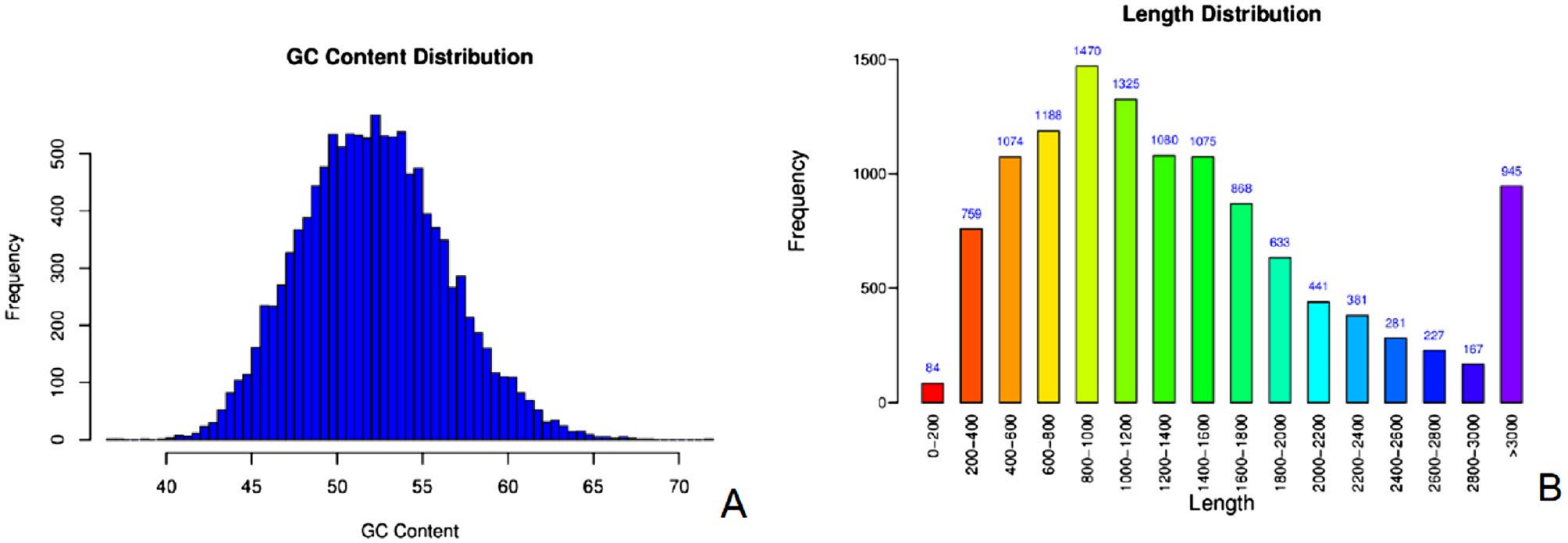
GC content distribution (**A**) and length distribution (**B**) of the whole genome of *Trichoderma polysporum* HZ-31. In (**A**), the horizontal axis indicates the GC content range, and the vertical axis indicates the number of predicted genes in each range. In (**B**), the horizontal axis indicates the length interval, and the vertical axis indicates the number of genes in each interval. Due to the wide range of sequence lengths above 3000 bp, they are combined.

GeneMark was used to predict the genes of the assembly results (Table 2). A total of 11,998 genes were predicted in the genome of *T. polysporum* HZ-31, and the total length of the genes was 17,908,516, which accounted for 45.54% of the length of the whole genome of *T. polysporum* HZ-31. The distribution map of gene lengths showed that the number of genes with lengths in the range of 800–1000 bp was the largest, including 1470 genes; and the number of genes with lengths of 0–200 bp was the smallest, including 84 genes (Fig. 1B). The distribution plot of GC contents of the genes showed that the GC content ranged from 45 to 55% (Fig. 1A), indicating that there was no significant bias in the GC content.

**Table 2:** Statistics of coding gene prediction results.

	All_num	> = 500 bp	> = 1000 bp	N50	Max_len	Min_len	All_len	Mean_len
Gene	11,998	10,619	7423	1809	69,120	121	17,908,516	1492.63

#### Repeat sequence prediction

The results of genome repeat sequence predictions of the sequenced strains are shown in Table 3. The results showed that *T. polysporum* HZ-31 contained 14,297 repeat sequences, with a total length of 1,149,690 bp, accounting for 2.94% of the total genome length. These sequences had an average length of 80.41 bp, of which 124 were DNA, 290 were LINE, 366 were LTR, 13 were SINE and 1610 were unknown.

**Table 3:** Repeat sequence statistics.

Repeat family	Regional count	Base count	Average length	Percentage in genome
DNA	124	42,407	341.99	0.11%
LINE	290	115,631	398.73	0.30%
LTR	366	120,692	329.76	0.31%
Low_complexity	1655	78,113	47.2	0.20%
SINE	13	1073	82.54	0.00%
Satellite	117	12,220	104.44	0.03%
Simple_repeat	10,122	393,372	38.86	1.01%
Unknown	1610	386,182	239.86	0.99%
All_RepeatSeq	14,297	1,149,690	80.41	2.94%

#### Non-coding RNA predictions

Non-coding RNAs are RNAs that do not code for proteins. Different strategies were used to predict the different non-coding RNAs with respect to their structural characteristics. An analysis of the results of the genomic data of *T. polysporum* HZ-31 revealed 148 transporter RNAs (tRNAs) and 45 ribosomal RNAs (rRNAs).

### Gene function annotation

The predicted protein sequences of the genes were compared with the functional databases, and the annotation results of the gene functional analysis are shown in Table 4. The numbers of annotated genes and the corresponding databases were: CDD 7418, KOG 5673, NR 11,541, PFAM 5818, SwissProt 7822, TrEMBL 11,531, GO 7983, and KEGG 3841.

**Table 4:** Gene function analysis annotation results.

Database	Number of unigenes	Percentage (%)
Annotated in CDD	7418	61.83
Annotated in KOG	5673	47.28
Annotated in NR	11,541	96.19
Annotated in PFAM	5818	48.49
Annotated in SwissProt	7822	65.19
Annotated in TrEMBL	11,531	96.11
Annotated in GO	7983	66.54
Annotated in KEGG	3841	32.01
Total unigenes	11,998	100

#### NR annotation results

**Figure 2:**
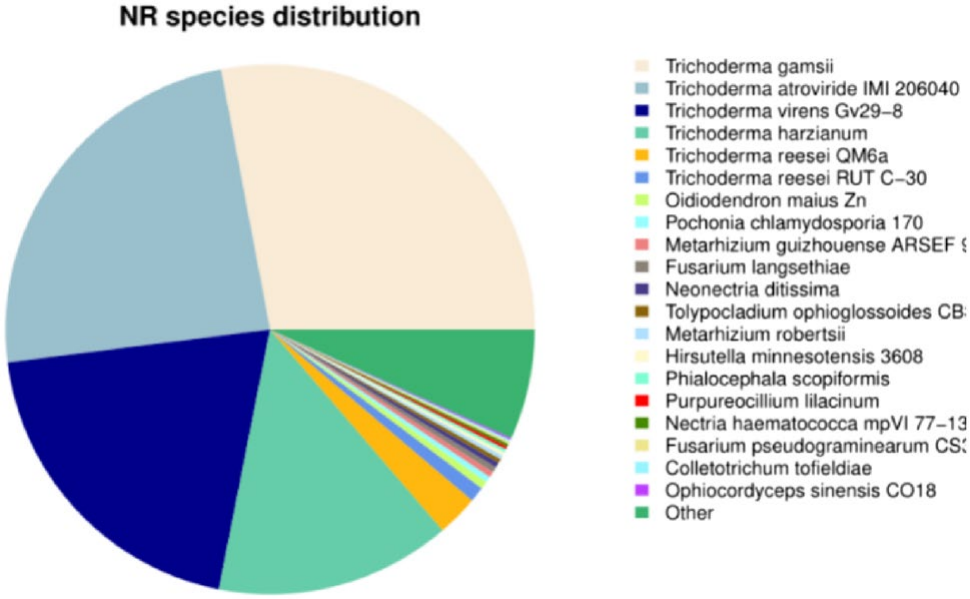
NR database species annotation statistical graph.Each sector represents a species, and the larger the sector, the greater the number of sequences aligned to that species.

Comparing the genomic genes of *T. polysporum* HZ-31 with the NR database (Fig. 2), a total of 10,360 genes were annotated to the genus *Trichoderma*. They accounted for 86.35% of the genome, indicating that strain HZ-31 indeed belongs to the genus *Trichoderma*. Among them, the most genes were annotated to *Trichoderma gamsii* with 3229, followed by *Trichoderma atroviride* with 2771.

#### KOG Functional classification annotation results

**Figure 3:**
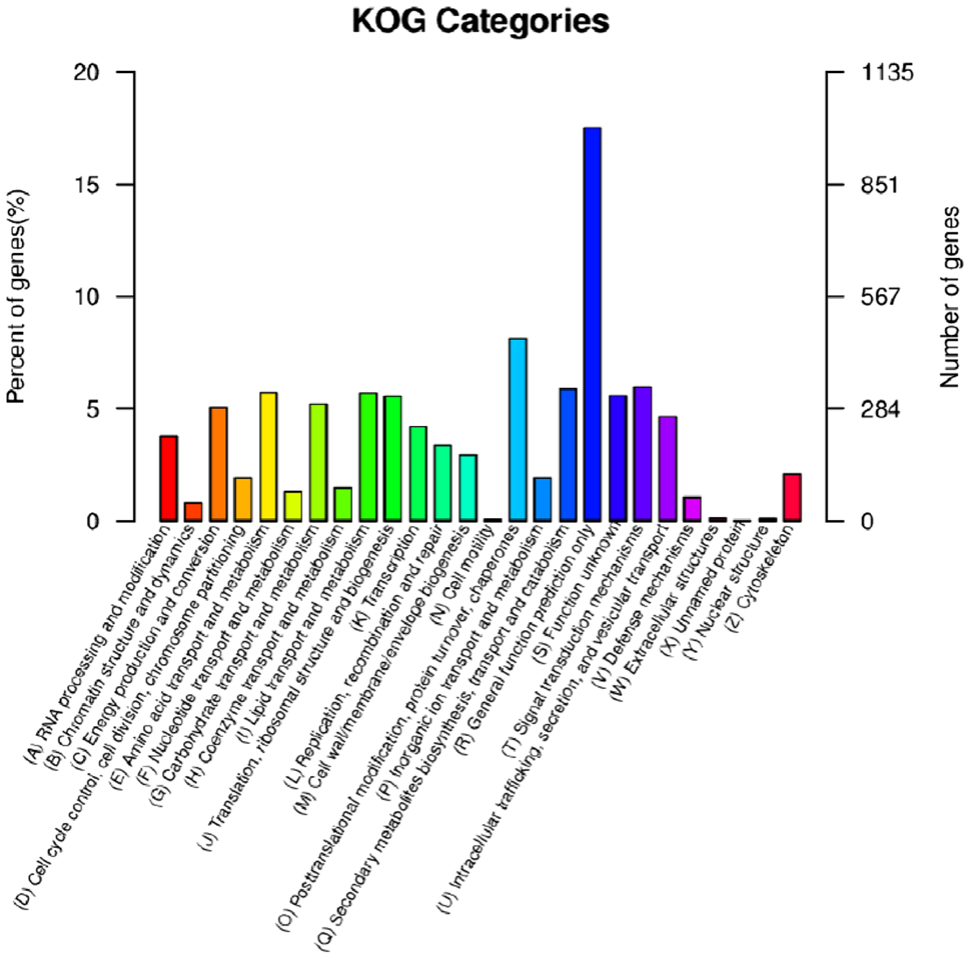
KOG categorical statistical chart.Each category on the horizontal axis represents a functional classification of a KOG, and the two vertical axes represent the percentage of genes (left) and the number of genes (right) annotated to each classification.

The genomic genes of *T. polysporum* HZ-31 were annotated to the KOG database (Table 5 and Fig. 3). The metabolic pathway with the highest number of annotated genes was General function prediction with 994; followed by Posttranslational modification, protein turnover, chaperones with 460; Signal transduction mechanisms with 338; and Secondary metabolites biosynthesis, transport and catabolism with 333.

**Table 5:** KOG functional classification statistics.

KOG functional classification	Gene_num	Gene_ratio
Processing and modification	214	3.77
Chromatin structure and dynamics	46	0.81
Energy production and conversion	286	5.04
Cell cycle control, cell division, chromosome partitioning	108	1.9
Amino acid transport and metabolism	324	5.71
Nucleotide transport and metabolism	73	1.29
Carbohydrate transport and metabolism	295	5.2
Coenzyme transport and metabolism	83	1.46
Lipid transport and metabolism	323	5.69
Translation, ribosomal structure and biogenesis	315	5.55
Transcription	239	4.21
Replication, recombination and repair	191	3.37
Cell wall/membrane/envelope biogenesis	167	2.94
Cell motility	3	0.05
Posttranslational modification, protein turnover, chaperones	460	8.11
Inorganic ion transport and metabolism	108	1.9
Secondary metabolites biosynthesis, transport and catabolism	333	5.87
General function prediction only	994	17.52
Function unknown	316	5.57
Signal transduction mechanisms	338	5.96
Intracellular trafficking, secretion, and vesicular transport	264	4.65
Defense mechanisms	62	1.09
Extracellular structures	6	0.11
Unnamed protein	1	0.02
Nuclear structure	5	0.09
Cytoskeleton	119	2.1

#### GO functional classification annotation results

**Figure 4:**
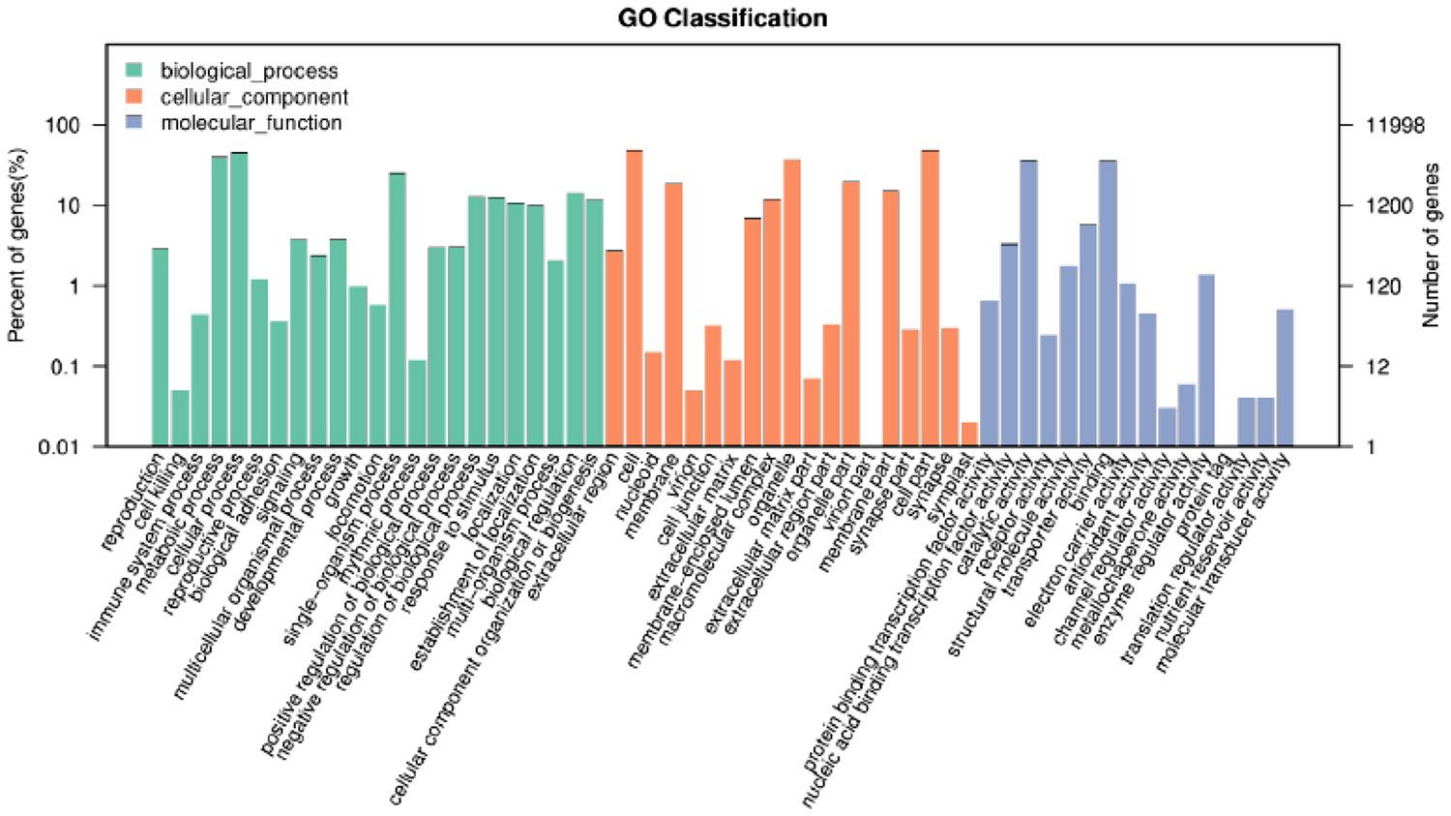
GO categorical statistics.The horizontal axis indicates the secondary classifications of GO, and the two vertical axes indicates the number of genes in each classification (right) and their percentage in the total number of annotated genes (left). Different colors represent different orthologs.

The predicted genes were categorized into cellular component, molecular function and biological process according to their functions in the GO database. The statistical results of gene functions and numbers of genes of *T. polysporum* HZ-31 annotated in the GO database are shown in Table 6 and Fig. 4. There were 25,064 genes belonging to cellular components, 19 categories; 10,570 genes belonging to molecular function, 16 categories; and 24,740 genes belonging to biological process, 23 categories. Among them, the most annotated genes in cellular components are in the category of cell, with 5713 genes; the most annotated genes in molecular functions are in catalytic activity, with 4429 genes; and the most annotated genes in biological processes are in cellular processes, with 5395 genes.

**Table 6:** GO functional classification statistics.

Ontology	Term	Gene_num	Ratio
Biological process	Reproduction	350	2.92
	Cell killing	6	0.05
	Immune system process	53	0.44
	Metabolic process	4789	39.91
	Cellular process	5395	44.97
	Reproductive process	143	1.19
	Biological adhesion	43	0.36
	Signaling	447	3.73
	Multicellular organismal process	287	2.39
	Developmental process	451	3.76
	Growth	118	0.98
	Locomotion	70	0.58
	Single-organism process	3027	25.23
	Rhythmic process	14	0.12
	Positive regulation of biological process	360	3
	Negative regulation of biological process	364	3.03
	Regulation of biological process	1511	12.59
	Response to stimulus	1492	12.44
	Localization	1290	10.75
	Establishment of localization	1188	9.9
	Multi-organism process	246	2.05
	Biological regulation	1701	14.18
	Cellular component organization or biogenesis	1395	11.63
Cellular component	Extracellular region	330	2.75
	Cell	5713	47.62
	Nucleoid	18	0.15
	Membrane	2222	18.52
	Virion	6	0.05
	Cell junction	38	0.32
	Extracellular matrix	14	0.12
	Membrane-enclosed lumen	815	6.79
	Macromolecular complex	1411	11.76
	Organelle	4475	37.3
	Extracellular matrix part	8	0.07
	Extracellular region part	40	0.33
	Organelle part	2370	19.75
	Virion part	1	0.01
	Membrane part	1822	15.19
	Synapse part	33	0.28
	Cell part	5710	47.59
	Synapse	36	0.3
	Symplast	2	0.02
Molecular function	Protein binding transcription factor activity	78	0.65
	Nucleic acid binding transcription factor activity	400	3.33
	Catalytic activity	4429	36.91
	Receptor activity	29	0.24
	Structural molecule activity	209	1.74
	Transporter activity	692	5.77
	Binding	4307	35.9
	Electron carrier activity	129	1.08
	Antioxidant activity	54	0.45
	Channel regulator activity	3	0.03
	Metallochaperone activity	7	0.06
	Enzyme regulator activity	162	1.35
	Protein tag	1	0.01
	Translation regulator activity	5	0.04
	Nutrient reservoir activity	5	0.04
	Molecular transducer activity	60	0.5

#### KEGG functional classification annotation results

**Figure 5:**
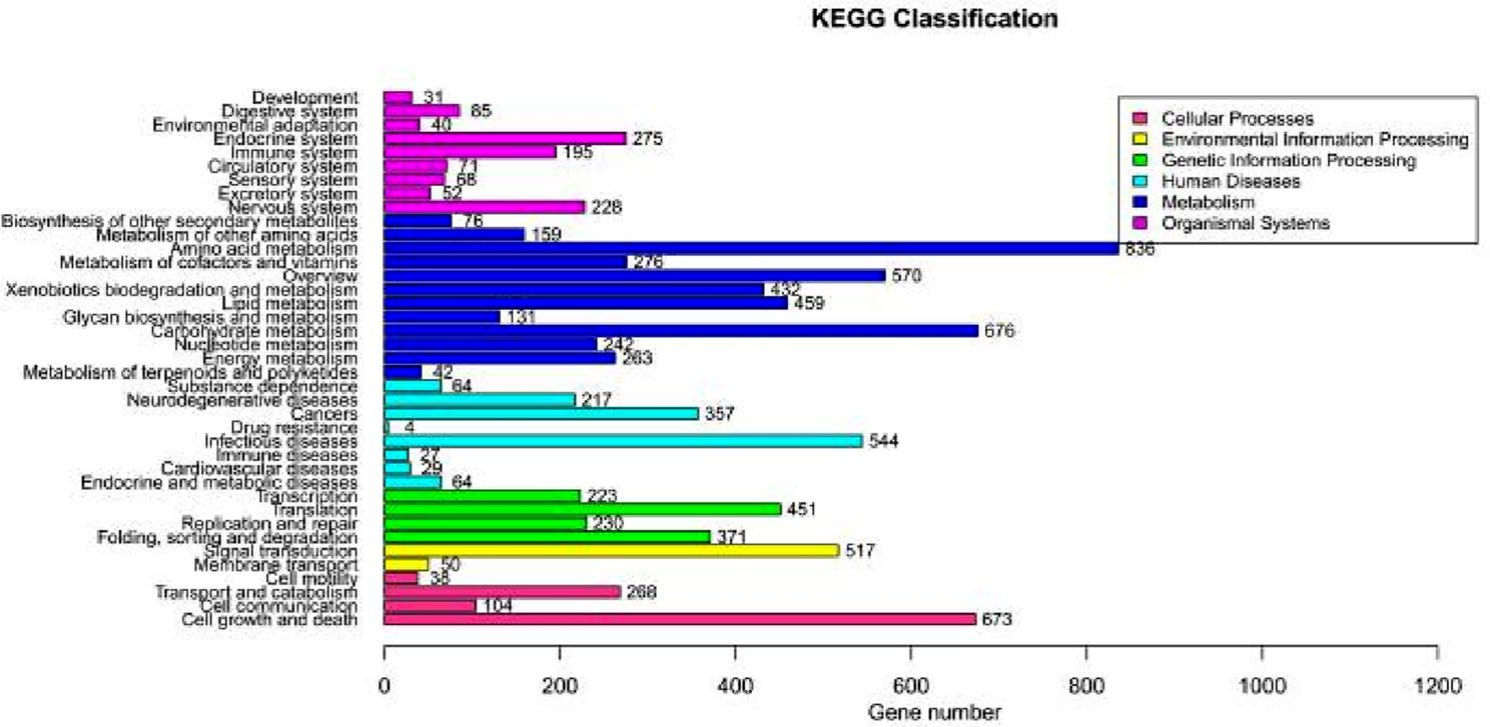
KEGG categorical statistics.The vertical axis indicates the name of each metabolic pathway involved, and the horizontal axis indicates the number of genes annotated to each pathway.

The genes of the *T. polysporum* HZ-31 genome were annotated to the KEGG database in six major categories (Table 7 and Fig. 5), including Cellular Processes, Environmental Information Processing, Genetic Information Processing, Human Diseases (HDP), Metabolism, and Organismal Systems, and they included 1083, 567, 1275, 1306, 4162, 1045 genes, respectively. Among these six categories, Metabolic processes had the most genes annotated, with Amino acid metabolism annotated to 836 genes; Carbohydrate metabolism annotated to 676 genes; Overview annotated to 570 genes; Lipid metabolism annotated to 459 genes; and Xenobiotics biodegradation and metabolism annotated to 432 genes.

**Table 7:** KEGG functional classification statistics.

Type	Subgroup	Gene_num
Organismal Systems	Nervous system	228
	Excretory system	52
	Sensory system	68
	Circulatory system	71
	Immune system	195
	Endocrine system	275
	Environmental adaptation	40
	Digestive system	85
	Development	31
Metabolism	Metabolism of terpenoids and polyketides	42
	Energy metabolism	263
	Nucleotide metabolism	242
	Carbohydrate metabolism	676
	Glycan biosynthesis and metabolism	131
	Lipid metabolism	459
	Xenobiotics biodegradation and metabolism	432
	Overview	570
	Metabolism of cofactors and vitamins	276
	Amino acid metabolism	836
	Metabolism of other amino acids	159
	Biosynthesis of other secondary metabolites	76
Human Diseases	Endocrine and metabolic diseases	64
	Cardiovascular diseases	29
	Immune diseases	27
	Infectious diseases	544
	Drug resistance	4
	Cancers	357
	Neurodegenerative diseases	217
	Substance dependence	64
Genetic Information Processing	Folding, sorting and degradation	371
	Replication and repair	230
	Translation	451
	Transcription	223
Environmental Information Processing	Membrane transport	50
	Signal transduction	517
Cellular Processes	Cell growth and death	673
	Cell communication	104
	Transport and catabolism	268
	Cell motility	38

### Analysis of secondary metabolite-related genes

#### Carbohydrate-active enzymes (CAZymes)

Phytopathogenic fungi secrete a variety of carbohydrate-active enzymes, which can be subdivided into different families according to their functions, such as Glycoside Hydrolases (GHs), Glycosyl Transferases (GTs), Polysaccharide Lyases (PLs), and Carbohydrate Esterases (CEs), Auxiliary Activities (AAs), and Carbohydrate-Binding Modules (CBMs)^17^.

**Figure 6:**
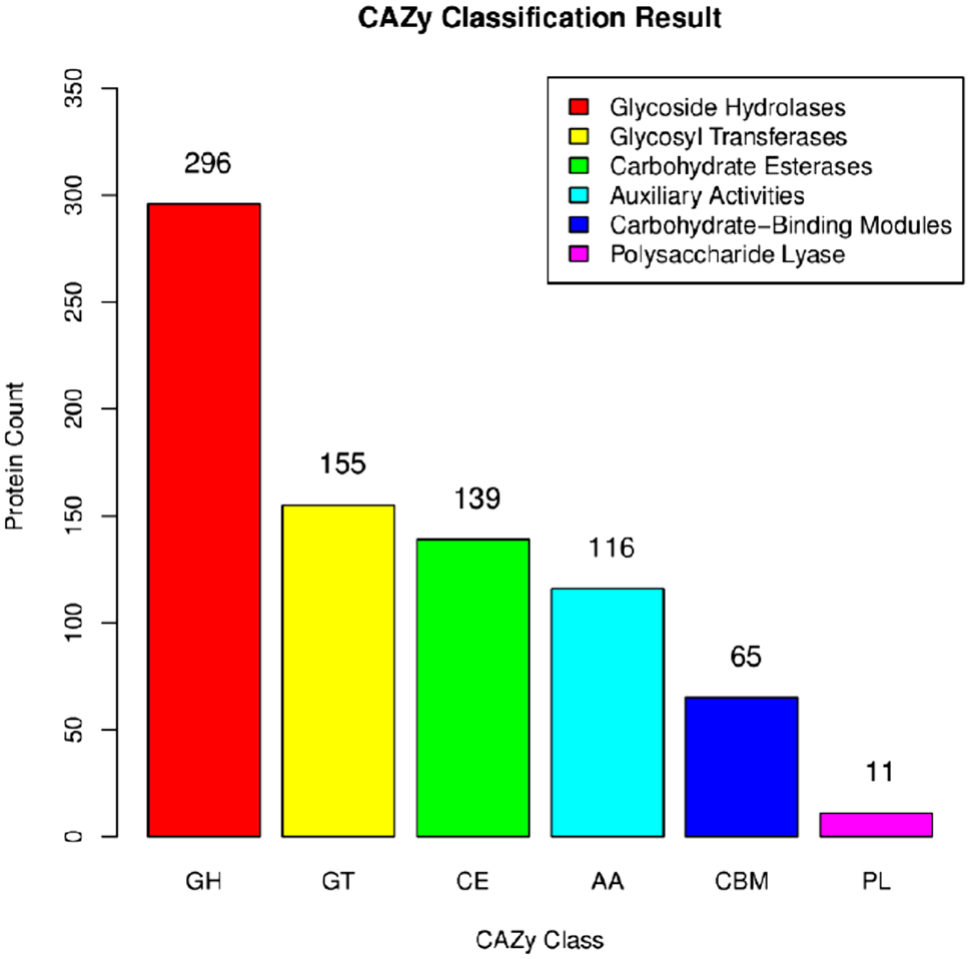
CAZy annotation of *Trichoderma polysporum* HZ-31 genes.The horizontal axis indicates the abbreviations of the six functional categories of CAZy, the vertical axis indicates the number of sequences contained in each functional category, and the figure legend gives the full names of the functional categories.

The protein encoding genes for *T. polysporum* HZ-31 were annotated to the CAZy database with a total of 782 genes (Fig. 6) The largest number of genes were annotated to the Glycoside hydrolase family with 296 (37.85%), while the smallest number of genes were annotated to the Polysaccharide cleavage enzyme family with 11 (1.41%). The remaining genes were annotated to glycosyltransferases, sugar esterolytic enzymes, oxidoreductases, and carbohydrate-binding structural domains, and numbered 155, 139, 116, and 65 genes, respectively, for percentages of 19.82%, 17.77%, 14.83%, and 8.31%, respectively. The greatest number genes annotated to the *T. polysporum* HZ-31GH family were the genes encoding GH18 with 35, followed by GH5, GH16, and GH3 with 21, 19, and 19, respectively. The most frequently annotated gene in the HZ-31GT family of *T. polysporum* were the genes encoding GT41, with 50 genes, followed by GT32, GT2, and GT21, with 12, 11, and 8 genes, respectively.

#### Secondary metabolic gene clusters

**Figure 7:**
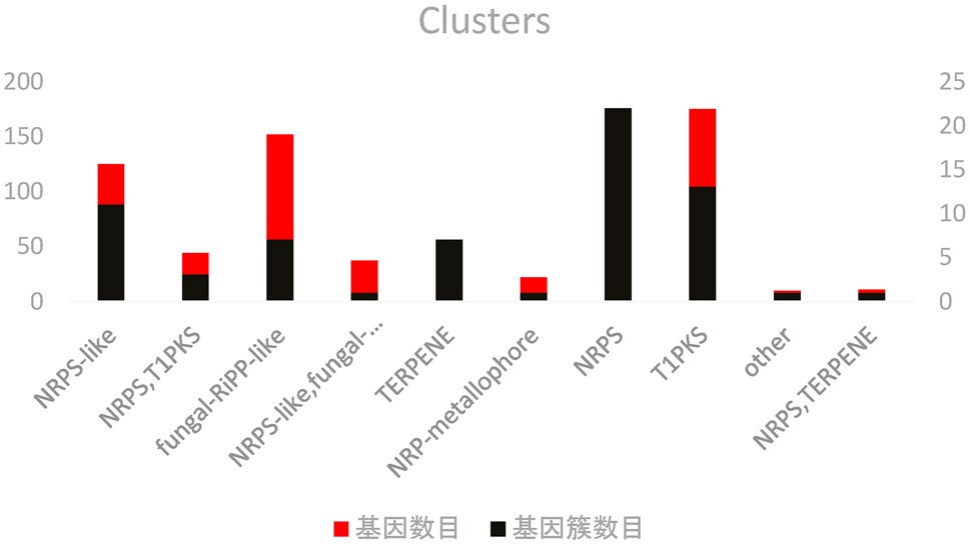
Secondary metabolite annotation of *Trichoderma polysporum* HZ-31.The horizontal axis indicates the gene cluster type, the left vertical axis indicates the number of genes, and the right vertical axis indicates the number of gene clusters.

Secondary metabolites are key factors in the phytotoxic activity of pathogenic fungi, and a variety of phytotoxic secondary metabolites, including polyketides, non-ribosomal peptides, terpenes, and alkaloids, are used to kill host cells. A total of 67 gene clusters were identified in the genome of *T. polysporum* HZ-31 (Fig. 7). The highest percentages were in the polyketide synthase gene clusters of type I (T1PKS), non-ribosomal peptide synthase-like gene clusters (NRPS-Like), peptide-like clusters synthesized and post-translationally modified in the fungal ribosome (fungal-RiPP-like), and non-ribosomal peptides (NRPS), and the highest percentage of terpenes (TERPENE), while NRP-metallophore accounted for less.

A BLAST comparison of all *T. polysporum* HZ-31 gene clusters with known secondary metabolite gene clusters revealed that the 1421 _g gene in the NRPS-Like, fungal-RiPP-like gene cluster type had 100% similarity to the cluster encoding choline. The 2916 _g and 2919 _g genes in the NRPS, T1PKS gene cluster type had 66% similarity to the cluster encoding *C. albicans* beauvericin. The 2916 _g and 2919 _g genes in the NRPS, T1PKS gene cluster type showed 66% similarity to the cluster of genes encoding beauvericin. The 5536 _g genes in the NRPS gene cluster type showed 100% similarity to the cluster of genes encoding verticillin. The 6364 _g genes in the NRPS gene cluster type showed 100% similarity to the cluster of genes encoding peramine/intermediate 1/intermediate 2. The 7085 _g genes in the NRPS gene cluster type showed 100% similarity to the gene cluster encoding enniatin, and the 7561 _g genes in the NRPS gene cluster type showed 100% similarity to the gene cluster encoding (-)-Mellein. There was 100% similarity between the 7905 _g gene in the T1PKS gene cluster type and the gene cluster encoding the epoxycyclohexanol-like novel natural product trichoxide. The 4736 _g gene in the NRPS-like, T1PKS gene cluster type showed 50% similarity to the gene cluster encoding swainsonine.

#### PHI pathogenicity related genes

**Figure 8:**
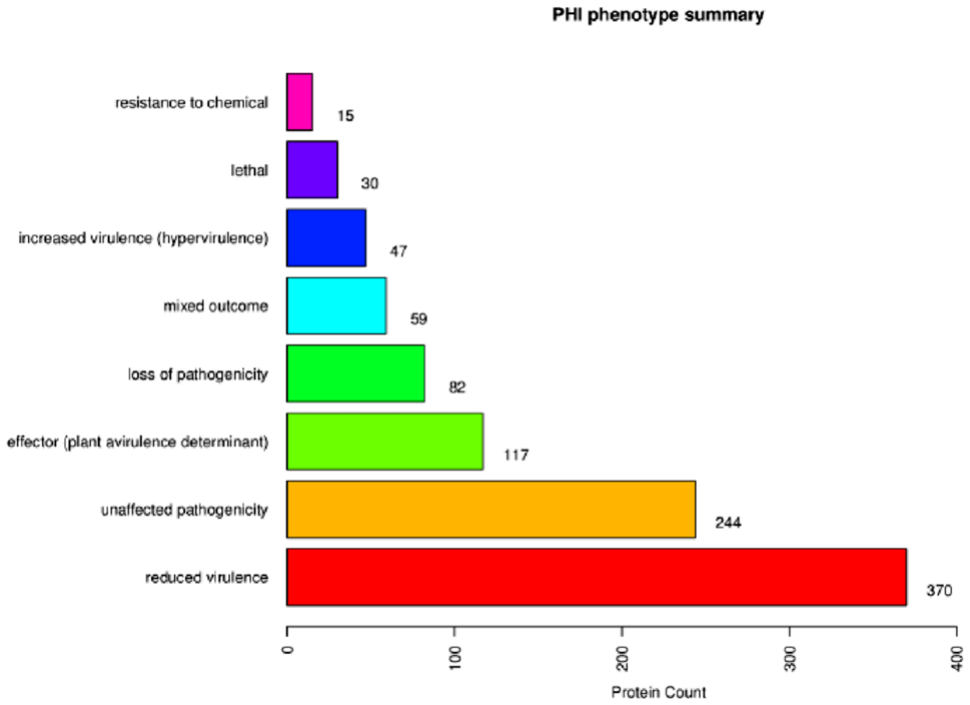
PHI annotation of *Trichoderma polysporum* HZ-31.The vertical axis represents the nine phenotypes of pathogen-host interactions, and the horizontal axis represents the number of genes annotated to each phenotype.

PHI annotation of the genomic genes of *T. polysporum* HZ-31 showed that a total of 757 genes were annotated in the database for pathogen-host interactions (Fig. 8). When the pathogen genes were functionally categorized, the highest number of genes were annotated as reduced virulence, 370; followed by unaffected pathogenicity, 244; loss largest of pathogenicity, 82; lethal, 30; and resistance, 30; chemistry target: resistance to chemical, 15; and effector: plant avirulence determinant, 117, A total of 47 were annotated as increased virulence, hypervirulence. Among them, the GPA1 gene, with a relatively high number of annotations in the Pathogen-Host Interaction Database (PHIDB), was numbered gene9200 in the genome of strain HZ-31, with a total length of 1062 bp. It encodes the G protein α subunit, which is related to the nutrient growth, sporulation, adherent cell formation, and toxin production of the fungus, and it is involved in the pathogenicity of *Cryptoccus neo formans, Aspergillus nidulans*, *Ustilago esculenta*, *Fusarium graminearum* and *T.harzianum.* The knockdown of the GPA1 gene could cause the complete loss of pathogenicity of *F. graminearum* GPA1 mutant on wheat spikes^18^. Further studies have also confirmed that the GPA1 gene can affect adherent cell formation and the expression of several virulence-related genes associated with infestation through the regulation of intracellular cAMP levels.

#### Signaling peptide proteins

A total of 1023 signal peptide proteins (8.53%) were predicted for *T. polysporum* HZ-31 (Table 8).

**Table 8:** Signal peptide protein prediction statistics.

Organism type	Total protein number	Signal proteins from SignalP-TM	Signal proteins from SignalP-noTM	Total signal proteins	Signal protein ratio (%)
euk	11,998	30	993	1023	8.526421

## Discussion

*Trichoderma polysporum* HZ-31 is a microbial fungus with great potential for weed control, but it is difficult to comprehensively analyze the mechanism of action of *T. polysporum* by traditional experimental and identification methods. In this regard, an in-depth study of the intrinsic causes of *T. polysporum* pathogenicity at the genomic level is of great significance. Therefore, we obtained the genome size of *T. polysporum* HZ-31 by whole genome sequencing and bioinformatics analysis as 39,325,746 bp, with 48% GC content, and the number of coding genes was 11,998. Among these genes, 148 tRNAs and 45 rRNAs were predicted in the annotated GO, COG and KEGG databases as related to amino acid metabolism, carbohydrate metabolism and lipid metabolism.

A variety of carbohydrate-active enzymes secreted by plant pathogens are involved in the degradation of host plant cell walls. Several studies have shown that pathogens from animals and plants utilize carbohydrases and other nutrients to regulate their virulence and adjust their metabolism for successful colonization^19^. A total of 782 genes were annotated in the carbohydrase database in this study. Members of the glycoside hydrolase family act as virulence factors and modulate plant immune responses during pathogen infection^20^. Among them, the gene encoding GH3 was annotated in *T. polysporum* HZ-31, and it was found to encode a β-glucosidase that plays an important role and is a key enzyme in cellulose degradation, which is closely related to the pathogen's infectious characteristics. Wang et al.^21^ found that the cell wall degrading enzyme of *Ziziphus jujuba melanogaster is β-glucosidase*, which plays a key role in the pathogenic process, is β-glucosidase, and its activity is the highest in the diseased-healthy junction during the process of infection. Li^22^ found 16 GH3 gene family members in the genome of *Aspergillus sphaericus*, and the transcripts of most of them were up-regulated under the induction of cellulose, which was consistent with the changes in extracellular β-glucosidase activity. Studies have suggested that the GH3 gene family in *Xylaria* plays an important role in cellulose degradation and plant pathogenicity.

Glycosylation is an important post-translational modification of proteins, which can affect the solubility, stability and catalytic activity of proteins, and it also has important biological functions related to protein folding, localization and translocation. In recent years, a growing number of studies have demonstrated that glycosyltransferases are closely related to pathogenic virulence and play key roles in biological processes such as the adhesion, immune escape and colonization of pathogenic bacteria. The gene encoding GT2 of the glycosyltransferase family in *T. polysporum* HZ-31 was annotated, and GT2 was shown to be involved not only in biomass synthesis, but also in many complex aspects of cellular processes in fungi. Zhang et al.^23^ used CRISPR/Cas9 and homologous recombination techniques involving deletion and backfilling of the *PaGt2* gene of the GT2 family encoding glycosyltransferase, and found that the resulting strain was significantly inhibited in nutrient growth, did not produce conidiophores and conidia, and had significantly reduced pathogenicity on peach shoots and fruits.

Genomics, molecular biological and bioinformatics studies have shown that the genes encoding enzymes which produce various fungal secondary metabolites are clustered and often in close proximity to telomeres^24^. The genes that are found in clusters of secondary metabolite synthesis genes are frequently co-regulated according to the functions of the secondary metabolites encoded by these genes^25^. Furthermore, an increasing number of secondary metabolite synthesis genes are thought to be closely related to, or even regulate, the pathogenicity of pathogenic bacteria. In this study, the *T. polysporum* HZ-31 secondary metabolite synthesis gene cluster was annotated to genes that synthesize toxins such as enniatin, beauvericin and Mellein. Beauvericin is a non-specific phytotoxin with toxic effects on many cell lines, and the essential components in its synthesis are the amino acids L-Phe, D-HYIV, ATP/Mg+ and ADOMet^26^. The mechanism of its cytotoxicity involves its role as a K^+^ ion carrier, in which it can be embedded in biological membranes where it forms channels, thereby triggering the elevation of Ca^2+^ in the cytoplasm, affecting the electrochemical gradient of the cell membrane, and ultimately inducing a series of cytotoxic reactions. Beauvericin can also enter the nucleus of plant cells, combine with DNA to form DNA-BEA complexes, and through calcium-dependent endonuclease cleavage of the bound DNA, it can interrupt chromosomes and cause toxicity. Chen^27^ found that knockdown of the leukocidin homologous gene *FOXB*_16250 in *Fusarium spinosum* Fo5176 resulted in a reduction in the pathogenicity of the Fo5176 mutant, as well as a delay in the onset of disease when the mutant was inoculated into Columbia-type *Arabidopsis thaliana* wild-type plants, suggesting that leukocidin synthesis genes inhibit *Fusarium spinosum* pathogenicity. Enniatin is a hexapeptide fungal toxin that is present in the mycelium that can have a strong toxic effect on the cellular tissues of plants [28]. The *ESYN1* gene is an important regulator of the biosynthesis of enniatin. Chen et al.^29^ cloned the *ESYN1* gene from Foc4, and compared with the wild strain, and found that the biosynthesis of enniatin in *Fusarium enantiospirillum* was significantly reduced in the knockout mutant. Furthermore, the pathogenicity of the mutant was completely lost, whereas backfilling of the wild strain was able to restore the enniatin biosynthesis and pathogenicity of *Fusarium enantiospirillum*, which suggests that this gene is a key factor in the pathogenic bacterial infections of the host plant. Mellein is a known compound with various phytotoxic, cytotoxic, fungicidal, antimicrobial and larvicidal activities^30^. Li et al.^31^ demonstrated the presence of (R)-(-)-mellein in the fermentation broth of *Vitis vinifera* and found that it was also present in *Vitis vinifera*-infected apple fruits and twigs, and that there was a relationship between lesion expansion and honey curdling mycorrhizal fungal pigmentation in the apple tissues. Phytotoxicity bioassays have shown that honeystrobin causes discoloration and death of apple leaves and browning of stems. Another study showed that the main components of the toxin of *Sphaeropsis sapinea*, the cause of pine tree dieback disease, are also the above two forms of R-(-)-Mellein and 4-hydroxyMellein, in which R-(-)-Mellein plays a major role, while the other two components are synergistic with each other in the toxic and antifungal activity assays^32^. These secondary metabolites play a key role in the pathogenicity of pathogens in their host plants, and the gene clusters that regulate the synthesis of secondary metabolites are fundamental in the regulation of that pathogenicity. The gene clusters of secondary metabolite synthesis annotated in the present study may also play important roles in the pathogenicity of *T. polysporum* HZ-31 in weeds.

By sequencing the whole genome of the *T. polysporum* HZ-31 strain, all the genetic information for the genome of this pathogen was obtained. Many virulence-related pathogenic genes were found, which were mainly involved in cell wall catabolic enzymes, strain nutrient growth, biomass synthesis, and other processes. The genome information also showed that *T. polysporum* HZ-31 contains a large number of genes involved in toxin biosynthesis, suggesting that *T. polysporum* HZ-31 is able to produce a variety of toxins during the infection process. The present study bridges the gap in the genomic information of this strain, and also provides the necessary genetic background information for further analyzing the herbicidal mechanism of this strain.

## Data Availability

The sequence data supporting the results of this study has been deposited NCBI with the main entry code PRJNA941260.
